# Health Policy on the pages of *Revista de Saúde Pública*

**DOI:** 10.1590/S1518-8787.2016050000180

**Published:** 2016-10-26

**Authors:** Aylene Bousquat, Oswaldo Yoshimi Tanaka

**Affiliations:** I Departamento de Prática de Saúde Pública. Faculdade de Saúde Pública. Universidade de São Paulo. São Paulo, SP, Brasil

**Keywords:** Health Policy, Planning and Management, trends, Publications, trends, Review, Historical Article

## Abstract

We carried out a narrative review of the scientific production in the area of Policy, Planning and Management in *Revista de Saúde Pública* (RSP), between 1967 and 2015. All the fascicles of RSP, in the period, were accessed via SciELO platform, which provides all articles online. We selected and classified the articles according to the main topics of scientific production in the area of Policy, Planning and Management. *Revista de Saúde Pública* has published 343 articles on this subject, with significant growth in the last two decades. The most discussed topics were Health Economics, Primary Health-care, Access and Use of Health Services, and Evaluation of Services and Programs. In the last decade, the topics of Policy and Access to Medicines and Public-Private Relationship, including judicialization, gained importance. The pages of RSP embraced the vast and diverse production of Policy, Planning and Management in its first 50 years, contributing to the consolidation of the area in Brazil.

## RESUMO

Foi realizada revisão narrativa da produção científica do campo de Política, Planejamento e Gestão veiculada na Revista de Saúde Pública (RSP), entre 1967 e 2015. Todos os fascículos da RSP, no período, foram acessados via plataforma SciELO, que disponibiliza todos os artigos *online*. Foram selecionados e classificados os artigos segundo os principais temas da produção científica da área de Política, Planejamento e Gestão. Foram publicados 343 artigos, com crescimento expressivo nas últimas duas décadas. Economia da Saúde, Atenção Primária à Saúde, Acesso e Utilização de Serviços de Saúde e Avaliação de Serviços e Programas foram os temas mais abordados. Na última década, os temas Política e Acesso a Medicamentos e Relação Público-Privado, incluindo a judicialização, ganharam importância. As páginas da RSP abrigaram vasta e diversa produção de Política, Planejamento e Gestão nos seus primeiros 50 anos, contribuindo para consolidação da área no Brasil.

## INTRODUCTION

In the first pages of *Revista de Saúde Pública* (RSP), a Rodolfo Mascarenhas’ article^58^ was published, addressing the funding of Public Health services vis-à-vis the national tax structure, an issue still present in the national scenario of the second decade of the 21st century. Since then, Brazilian and Latin American Health Policies have occupied a prominent place.

The period of emergence of RSP coincides with the international consolidation of the field of study of Public Policies, Policy Science or Policy Studies, which in Brazil was named Public Policy Analysis, among them those of Health^96^. The analysis of Health Policies incorporate issues both related to power (politics) and those which lay down guidelines, plans, and programs (policy)^75^. The organization of practices and ways of management also make up the field of Policy, Planning and Management (PPM), one of the constitutive axes of Brazilian Public Health^73^ ^,^ ^92^.

In the last 50 years, Brazil has substantively changed its demographic, social, political, and economic profile. The Brazilian health system also changed: the meritocratic model, with access conditioned to formal labor market integration, was replaced by a universal system with the creation of the Brazilian Unified Health System (SUS). The achievements and challenges faced in that process were countless. However, despite the significant increase in the access to health services, ensuring universality and integrality is still a challenge^76^. The interwoven and complex public-private relationships in the sector, aligned to a chronic under-financing with predominance of private spending, are also key elements in the contemporary discussion of the area of PPM.

The great changes described in the paragraph above can be read in the pages of RSP published in the past 50 years, as well as the several discussions by researchers of the area. Nothing more appropriate than, on the occasion of the 50th anniversary of RSP, revisiting all this period. Thus, this study aimed to describe the articles on PPM published between 1967 and 2015 in RSP.

## METHODS

In this study of narrative review, all fascicles of RSP were accessed via SciELO platform, which provides all articles online. The articles were selected and classified according to the main topics of the scientific production in the area of PPM, listed previously in researches conducted by Levcovitz et al.^49^, Paim and Teixeira^75^, Viana and Baptista^96^, and Teixeira^92^.

We included all published articles, classified as: original articles, special articles, comments, notes, updates, and reviews. We also analyzed Institutional Technical Notes and, more recently, articles published in the section of Public Health Practice.

## RESULTS

In the pages of RSP of the last 50 years, 343 articles were published in the area of PPM. During this period, RSP has gone from biannual publication in the first five years, to quarterly in the nine subsequent years, and bimonthly and in the last 36 years. Approaches with quantitative methods were the most common ones; the topics directly related to power issues in the analysis of Health Policies (Politics) were published more rarely.

A cross-sectional look over the five decades of RSP shows the permanence of some topics. The most covered ones were: Economics of Health, Primary Health-care, Access and Use of Health Services, and Evaluation of Services and Programs. More recently, the topics of Policy and Access to Medicines and Public-Private Relationship, including judicialization, gained importance.

The evolution of the number of articles published over the decades (blocks) and triennia (row) can be viewed in the Figure. Since the year 2000, we observe a trend of gradual increase in publication of articles, following the growth trend of the scientific production in the area of PPM in Brazil^92^ ^,^ ^96^. Two editorial practices contributed to greater publication of articles of this area: the Institutional Technical Reports and the Public Health Practice Section in 2013. The Technical Reports were prepared by different departments of the Ministry of Health or by State Departments of Health, to promote their Health Programs and Policies. The publication of Technical Reports contributed to consolidate RSP as an important space of discussion in the area of PPM. The Public Health Practice section, with support from the Department of Science and Technology of the Ministry of Health, aims to highlight the articles with more immediate application for planning, implementation, and evaluation of Health Policies, contributing to strengthen SUS. These two moments show the tradition and vocation of RSP to disseminate “knowledge that have the possibility to be effectively incorporated into the actions of public health policy, resulting in improvements to population health”^10^ (pag. 1).

**Figure:**
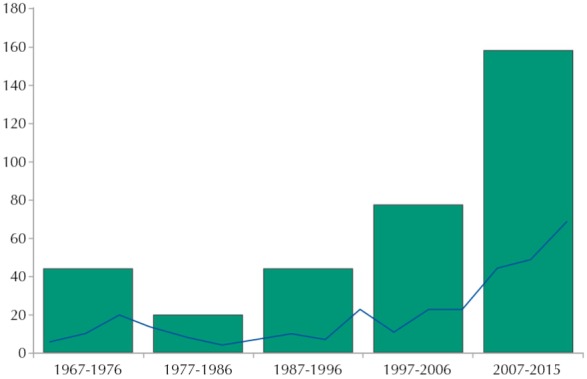
Number of articles in the area of Policy, Planning and Management, according to decades (blocks) and triennia (row). *Revista de Saúde Pública*, 1967-2015.

From 1967 to 1976, 44 articles that talked about the national and state political scenario were published. João Yunes’ studies^99^ ^-^ ^101^ are considered pioneers of the construction of Brazilian sanitary thought^16^. His articles characterize the offer of the so-called health services in the State of São Paulo and present the guidelines of an important administrative reform of the State Department of Health of São Paulo^29^. Some of the topics listed in the diagnostics (location and uneven distribution of services; deficient quality of care; insufficient resources) are still challenges to be overcome in the Brazil of the 21st century.

The provision of ambulatory and hospital health services to the welfare population, by the National Institute of Social Security, has been discussed in the articles by Lima Gonçalves et al.^51^ ^,^ ^52^, aligning RSP to an important political and academic debate of the period. They call attention to the publication of articles with topics and approaches characteristic of Health Economics, which was not usual in discussions of the area^6^ ^,^ ^7^.

The 1980s were a moment of production growth in the area of PPM: proposals for reform of the sector in the Country; the end of the military dictatorship; the establishment of several local health systems; and the proposal for the creation of SUS inspired several studies^96^. However, the second decade of RSP (1977-1986) had the lowest publication of articles of the area, with only 20 publications. The topics covered in this period were quite different (Primary Health-care [PHC], hospital administration, health services organization, funding, planning, among others). In the second decade of RSP, the articles of Maria Lúcia Lebrão^47^ and Carlyle Guerra de Macedo^54^ are worth mentioning. Lebrão assessed the feasibility of application of the International Classification of Appointment Reasons at PHC services in Brazil, an important topic little explored until now^46^. The presentation of Macedo in the fiftieth anniversary of Faculdade de Saúde Pública of Universidade de São Paulo was appropriately transcribed^54^. The author presents an accurate diagnosis on the situation of the health sector before the global economic crisis of the 1980s and the growth of neoliberal proposals for the sector, stating that : “what concerns us most in this crisis is not so much the characteristics of the crisis itself, but, above all, the solutions intended to be applied for overcoming it” (p. 68). The dramatic consequences for the population of a policy of fiscal adjustment and cuts in social areas are discussed, and unfortunately the current political and economic situation of the Country allows some of these excerpts to become relevant again.

Between 1987 and 1996, 44 articles were published in the area of PPM. The main topics discussed were Health Economics^8^ ^,^ ^41^ ^,^ ^42^ ^,^ ^77^ ^,^ ^82^ and Evaluation of Services, especially of PHC^40^ ^,^ ^83^. The overview of Health Policies in Brazil and in the State of São Paulo was also studied^79^ ^,^ ^91^. Dialoguing with the political context of the time, strongly marked by the creation of SUS, Sueli Dallari^26^ ^,^ ^27^ discusses important concepts to Brazilian Public Health: the right to health and equality, in line with the ongoing decentralization process. Sonia Fleury^32^ discusses the Social Protection models in Latin America and social inequities, a classic study of the relations between State, Social Protection Policies, and Health.

Teixeira^92^ identifies that, in the field of Public Health, the area of PPM has developed an important dialogue both with Epidemiology and Social Sciences. In RSP, the dialogue with Epidemiology was very fruitful and expressed itself in the several studies that examined the use and access to health services, especially by population-based surveys. Cesar et al.^19^ and Costa and Fachinni^24^ were pioneers in the use of population-based surveys for this purpose. Naturally, this approach was consolidated as an important tool to analyze and provide subsidies for the formulation and implementation of health policies.

The field of PPM has increased significantly in the last years of the 20th century and in early 21st century, with progressive inclusion of topics of interest and new theoretical approaches^92^ ^,^ ^96^. In the last 20 years, many topics have been published in the pages of RSP, such as: intergovernmental relations in the backdrop of Brazilian federalism^55^; health policies and equality assurance^4^ ^,^ ^17^; decentralization^1^ ^,^ ^9^ and regionalization of health services^28^; regional governance^85^; use of information for decision-making^23^; private health sector^12^ ^,^ ^14^ ^,^ ^44^ ^,^ ^50^ ^,^ ^80^ ^,^ ^94^; and public-private relations^71^ ^,^ ^81^.

During the same period, the Institutional Technical Reports presented central topics of health policies: Family Health Program^62^; Health Promotion^60^ ^,^ ^61^; Evaluation of incorporation of technologies^63^; Quality of hospital services^5^; National Drug Policy^88^; among others.

Specific policies were an important topic in RSP, especially Oral Health^4^ ^,^ ^65^ ^,^ ^87^. Mental Health^66^ ^,^ ^72^ and Urgent Medical Aid Service (SAMU)^56^ were also discussed. Surely, the articles focused on PHC and, more recently, on Family Health Strategy, were frequent in all 50 years of RSP, with special increase in the last two, reflecting the political and academic weight of this proposal for PHC implementation in the Country. The access and use of PHC services stood out in the publications of RSP^15^ ^,^ ^31^ ^,^ ^37^. Other themes also composed the studies on PHC, as Innovative Strategies^73^, humanization practices^68^, integrative practices^3^ ^,^ ^86^, and the performance of Community Health Agents in Oral Health promotion^33^. More recently, important issues were discussed, e.g., the Family Health Support Unit^45^ and the institutional support and matrix-based strategies^84^.

In recent years, RSP has started to address the Policy and Access to Medicines, an issue that lies at the center of agendas of the most distinguished health systems around the world. The article by Granjeiro et al.^38^ analyzed the financial sustainability of the policy of access to antiretroviral drugs. The authors indicated the need to strengthen the national productive sector, with emphasis on drugs not protected by patents. On the other hand, the access and use of medicines have been studied in the most diverse scenarios: older adults in urban area of the Northeast region^21^; people with disabilities in São Paulo^18^; *quilombola* population^59^; and in Central America^30^. The studies also approached the following topics: Pharmaceutical Assistance in the PHC scenario^67^; prices and availability of medicines in the Popular Pharmacy Program of Brazil^79^; and Policy of Medication Price Regulation^64^.

The access to medicines has interface with two central topics in the analysis of health policies in the 21st century: judicialization and evaluation of technological incorporation, which will be highlighted below.

SUS has been affected by increasing litigations that, if on the one hand make explicit the overlapping between health and juridical systems in Brazil, on the other, refer to the issues of equality and of the relation between individual and collective rights. The articles of Marques and Dallari^57^ and Vieira^98^ can be considered milestones in that field. Subsequently, several articles on legal demands for medications in many States of Brazil^22^ ^,^ ^53^ ^,^ ^90^ were published. The relations between the productive sector of medications with high technological incorporation and the legal system were unveiled by Chieffi and Barata^20^, which identified that a small number of lawyers answered by most of the lawsuits filed against SUS.

Articles on the evaluation of medicines acquisition, from cost effectiveness studies, were published recently on RSP^2^ ^,^ ^39^ ^,^ ^70^. This approach is one of those used by Health Economics, topic that gathered the greatest number of articles in the area of PPM in these first five decades of RSP. It is worth recalling that economic theories have been increasingly introduced in the analyses of health policies since the 1960s and 1970s. The crisis of modern social protection systems boosted even more these approaches. The topics commonly approached by Health Economics are: supply and demand, macroeconomics, funding and resource allocation, and economic evaluation; all present in RSP since its first issue^8^ ^,^ ^13^ ^,^ ^35^ ^,^ ^41^ ^,^ ^43^ ^,^ ^48^ ^,^ ^97^.

More recently, several criticisms have been made to the more formalist character of Health Economics. Proposals have been developed to discuss health from its specificities as commodity and economic good, as social right and space of capital accumulation, and the contradictions arising therefrom^95^. From this new perspective, the decommodification of access, commodification of offer, formation of the health economic-industrial complex, and articulations between Health and Development, as well as innovations, become central issues. Several articles approached these topics, especially in the last decade of RSP^11^ ^,^ ^25^ ^,^ ^34^ ^,^ ^35^ ^,^ ^69^ ^,^ ^89^ ^,^ ^93^, even with the publication of a special supplement on Health and Development, in 2012.

### Final Considerations

The pages of RSP sheltered a wide and diverse production of PPM in its 50 years, contributing to the consolidation of the area in Brazil. The academic community and policy makers found, in RSP, a place for fraternal debate, for discussion of the main policy proposals for health systems and services in Brazil and Latin America, for new methodological approaches, and for introducing new topics of interest.

The goal of Brazilian Public Health of building a universal health system in a country with immense social inequalities as Brazil was reflected in many of the lines published in RSP. The challenges and obstacles of this goal have been the subject of increasingly frequent reflection on RSP issues.

In its 50 years of trajectory, RSP showed its commitment to the principles of SUS and to the improvement of living and health conditions of more than 200 million Brazilians. The area of PPM has many challenges; among them, the study of the complex political and economic relations of the Brazilian health system is an eternally unfinished task, which will surely continue in the next pages of RSP.
